# Scholarship in Emergency Medicine: A Primer for Junior Academics Part I: Writing and Publishing

**DOI:** 10.5811/westjem.2018.39283

**Published:** 2018-10-18

**Authors:** Michael Gottlieb, Shahram Lotfipour, Linda Murphy, Chadd K. Kraus, James R. Langabeer, Mark I. Langdorf

**Affiliations:** * Rush University Medical Center, Department of Emergency Medicine, Chicago, Illinois; † University of California Irvine Health School of Medicine, Department of Emergency Medicine, Irvine, California; ‡ University of California Irvine, UCI Science Library Reference Department, Irvine, California; § Geisinger Health System, Department of Emergency Medicine, Danville, Pennsylvania; ¶ University of Texas McGovern School of Medicine, Houston, Texas

## Abstract

The landscape of scholarly writing, publishing, and university promotion can be complex and challenging. Mentorship may be limited. To be successful it is important to understand the key components of writing and publishing. In this article, we provide expert consensus recommendations on four key challenges faced by junior faculty: writing the paper; selecting contributors and the importance of authorship order; journal selection and indexing; and responding to critiques. After reviewing this paper, the reader should have an enhanced understanding of these challenges and strategies to successfully address them.

## INTRODUCTION

Writing and publishing are an important component of academic medicine. However, it can be challenging for many junior academicians to navigate the process to a successful publication. In fact, studies have consistently demonstrated that less than half of all conference abstracts are ever published as full manuscripts.^1^–^3^ Additionally, while many young researchers may benefit from local mentors guiding them through the authorship process, mentorship may be limited in many academic emergency medicine (EM) programs.^4^–^6^ After many years of navigating this process at various research universities, the authors concluded that a practical primer would be useful for residents, fellows, and junior faculty in EM. In addition, the advent of open-access publishing as an alternative to traditional subscription-based publishing expands the possibilities and perils of scientific communication.^7^ This is the first in a series of papers seeking to help faculty members and researchers maximize their scholarly efforts to develop their academic careers. In this article, we sought to incorporate expert consensus recommendations on improving scholarship in EM. This paper focuses on four common challenges faced by researchers when writing and publishing their academic work.

## WRITING YOUR ARTICLE

One of the biggest challenges to publishing is often writing the manuscript. After a study has been completed, the next step is to create the manuscript and submit for publication. Often, this can be facilitated by writing the introduction and methods sections prior to completing the study and finishing the results and discussion sections after completion, so that the burden of writing is less to overcome. Additionally, reading and peer reviewing other articles can be incredibly valuable by providing experience and insights into the scientific literature, as well as learning what features make a high-quality submission. It may be particularly useful to review several articles from the intended journal prior to submission to ensure that your style and language are consistent with prior accepted submissions. All journals also have authorship instructions, which include guidelines on formatting, section categories, and article limits (e.g., maximum figures, tables, references, word count). Authors should review these carefully and diligently to ensure that they completely follow all of the rules.

When writing a manuscript, it is important to follow a structure. The most common format is: abstract, introduction, methods, results, discussion, limitations, and conclusion. The introduction should be formatted such that it presents a summary of the literature and how the study fits into the current understanding of the topic. This has been referred to as the problem/gap/hook heuristic.^8^ In this model, Lingard suggests that an introduction must do three things: identify a problem of significance to the reader; establish a gap in the current knowledge or understanding of the problem; and articulate a hook that convinces the reader of the importance of this.^8^ The last sentence of the introduction commonly includes the research hypothesis and study aim. Authors should also keep the target audience in mind and ensure that the paper is specific and relevant to this group.

The methods section should clearly define the study protocol, such that it could be easily repeated by another investigator. Authors are advised to ensure that the population, intervention, control, outcome, and time interval are explicitly described.^9^,^10^ Authors should also review the Enhancing the Quality and Transparency of Health Research (EQUATOR) guidelines (http://www.equator-network.org/) for their specific study design and ensure that their manuscript addresses all of the reporting criteria. For example, if the authors are publishing an observational study, they should adhere to the Strengthening the Reporting of Observational Studies in Epidemiology (STROBE) guidelines,^11^ while if they are performing a randomized controlled trial, they should use the Consolidated Standards of Reporting Trials (CONSORT) criteria.^12^ These can also be valuable to help scaffold the paper and prevent writer’s block.

The results section should describe the study population, adherence to the protocol, and all relevant outcomes. It may be advantageous to include data in tables and figures to avoid an overly lengthy results section. One common pitfall is to repeat the results in both the tables and figures, as well as the text. Often, only one is necessary and tables and figures are generally preferable. Another common error is to discuss the significance of the findings in the results section. Any discussion of importance and relevance should be deferred to the discussion section.

The discussion section should focus on applying the results in the context of the current literature, including how it supports or refutes prior studies and how this will impact future patient care and research. The limitations section should address all potential biases and confines of the current study. All studies have limitations and it is important to address them as thoroughly as possible, both with respect to the potential influence on results and directions for future study.^13^ The last part of the discussion section (or formal conclusion section, if applicable) typically summarizes the authors’ conclusions and provides directions for future research.

Prior to submission, it is valuable to have a local colleague pre-review the paper and provide comments and feedback. This can help identify some of the sentence structure and grammatical errors,^14^–^16^ as well as provide an external opinion to ensure that the manuscript’s argument is persuasive and coherent.^17^,^18^ Bordage evaluated reasons why manuscripts were commonly rejected in a seminal paper in *Academic Medicine* (Table 1).^19^ Authors can avoid many of these common pitfalls by involving a statistician early in the project (preferably in the study design stages before the project has launched) to ensure that the methodology is appropriate for the study.

Along with the manuscript, most journals also require a cover letter and title page. The cover letter should include a brief summary of the proposed study and why it is important to the journal’s readership. The cover letter should also include how the study or results align with the journal’s mission statement. Many journals require specific components within the cover letter, which can include a statement of conflicts of interest or funding, so one should ensure that this is also included if required. The title page requirements can vary between journals, but most commonly include a listing of the authors and their affiliations, the contact author, keywords, word count, funding, and prior presentations of the research. Those who are interested in learning more should review the following resource: https://www.aliem.com/2017/11/template-journal-manuscript.

## SELECTING CONTRIBUTORS AND THE IMPORTANCE OF AUTHORSHIP ORDER

Authorship of publications is important for several reasons. Being designated as an author confers not only credit, but also responsibility for the findings and conclusions of the publication.^20^,^21^ While there are often more people involved in a research project than listed on the author block, only those who contribute substantially to the paper should receive authorship credit.^21^–^23^ The remainder may be included as an acknowledgment at the end of the paper, but should not be included as authors. Most experts recommend using the International Committee of Medical Journal Editors guideline to define authorship criteria (Table 2).^20^,^24^,^25^

Once you have decided on the author list, the next challenge is to determine the author order. The first author should be the person who contributed the most to the manuscript and receives the largest portion of the credit.^20^,^24^,^26^ The last author is often the senior author and typically receives similar credit to the first author, as that person is assumed to be the intellectual and financial resource for the research project.^24^,^26^

The remaining author order can vary significantly depending upon the authorship team and the type of research project. Unfortunately, this can create challenges, as not all authors receive equal credit by promotion and tenure committees, with some committees ascribing greater credit to the second author than all other subsequent authors listed after the first author.^20^ Some journals (more often with case report and review articles) will limit the number of authors for a manuscript, which can be important as you consider your author list. Additionally, many journals will limit the number of authors listed in the references to either three or six authors, followed by “et al.”, which can leave the remaining authors feeling more hidden with respect to recognition for the paper.^20^

There are several well-described, authorship sequencing strategies in the literature. The “sequence-determines-credit” approach is based upon the principle that each successive person after the first author contributed a progressively smaller portion to the manuscript.^20^,^26^ While this provides a simple mechanism for determining the author order, it is important to clearly explain to the other authors why each person is located in the specific location to avoid ill feelings between authors. Another strategy is referred to as the “equal contribution” approach. With this technique, all authors are given equal credit for the manuscript.^26^,^27^ Typically, all authors will be listed alphabetically by last name. This strategy may be preferable when the authors have contributed similar degrees of effort to the paper. There are also some variations among these techniques, wherein the first or last author are maintained as primary authors, while the remainder are listed alphabetically. In the medical field it is more common to follow the “sequence-determines-credit” approach, while in other scientific fields the “equal contribution” approach is more common. At the time of application for academic promotion, many research-oriented universities ask the candidate to declare the percentage of contribution effort for each published manuscript claimed during the review period. This gives you the opportunity to self-describe your role and effort.

Whichever strategy is selected, it is advised to discuss the author order early in the development of the paper to ensure that all parties are aware of and agree with the decision.^20^,^24^,^28^,^29^ However, you should allow room for flexibility, especially with respect to the middle authors, as the level of contributions may change over the course of the project. Typically, the first or last author will initiate the authorship conversation, but the other authors should also feel comfortable discussing this with the first author and study group.^28^,^29^

A separate role within the authorship block is the corresponding author, which is most commonly the first or last author. When the first author is a resident or a student, the corresponding author is often the senior author. The corresponding author is responsible for all publication correspondence regarding the article, both with respect to the journal itself and future readers. The corresponding author will be contacted by readers with questions regarding the research, requests for copyright release (with open-access journals), and could be challenged by other researchers to verify the methodology, statistics, or research results. While this is almost always the first or last author, it could be awarded to a different author to properly credit that person when she or he provided a substantial contribution to the project, but was not selected as the first or last author (e.g., originator of the project idea, the “second” senior author).^29^,^30^ Another approach could be dual first authors who are listed as first and second but have an asterisk with their names explaining the designation as dual first authors. It is important to note that some journals do not allow dual first-author designations.

Finally, it is important to discuss the importance of unique author identification. While researchers and readers are often able to easily distinguish the work of authors with uncommon surnames from others, readers can struggle to differentiate the work of authors from others sharing a similar surname and first initial.^31^ One technique to differentiate yourself is to add your middle initial to the author listing, decreasing the likelihood of ambiguity in article identification.^14^ An additional and more effective way is to apply for an Open Researcher and Contributor Identification (ORCID) account (https://orcid.org/).^31^ This is a non-profit organization that creates unique identifiers for researchers and is used by several publishers to help recognize authors for their work. Increasingly, journals and publishers are now requiring authors to include ORCID numbers during manuscript submission. This may also be valuable if the author undergoes a name change, as PubMed will not change or link your current name with your prior publications. Obtaining an ORCID is free and takes only a few minutes to accomplish.

## JOURNAL SELECTION AND JOURNAL INDEXING

There are a myriad of journals to which you could submit your research papers. To promote yourself and career, it is vital to understand the hierarchy of the quality and selectivity of journals. There are currently 78 journal titles that relate to EM in the Scimago Journal and Country Rank index (SJR). You can find an updated list at: http://www.scimagojr.com/journalrank.php?area=2700&category=2711. The supplemental table includes a list of the legitimate EM journals recognized by SJR and are indexed in Scopus as of this publication. An updated version of the list, maintained by the *Western Journal of Emergency Medicine* is available here: https://escholarship.org/uc/item/4pc1v507#supplemental.

A journal’s scope of indexing determines how another physician can find your paper to read and possibly cite. The supplemental table includes whether a title is indexed in each of the following databases: PubMed, PubMed Central (PMC), MEDLINE, and Clarivate (formerly Thomson-Reuters) Web of Science Expanded or Emerging Sources. These are the key life-science databases in which journals attempt to index their contents. It also includes whether a journal is fully open access, and both the SJR and Clarivate two-year impact factors (if available). Articles are ranked in order from highest to lowest SJR impact factor to assist with determining journal submission decisions. In general, the higher the impact factor, the more selective the journal is for accepting your submission. If a journal is *not* listed, the quality of the journal may be questionable. For newer journals, it can be valuable to review the list of accepted publications to determine the quality of submissions. Discussing with more experienced researchers and medical librarians can also be valuable for assessing the potential quality of the journal.

Deciding where to submit may be overwhelming to more novice researchers. While it may seem tempting to submit to the journal with the top impact factor or a familiar journal title, it is important to select an appropriate journal to have the best chance of acceptance. You should begin by determining whether the journal accepts the category of article you are planning to submit. For example, while the *Western Journal of Emergency Medicine* no longer accepts case reports, its affiliated journal *Clinical Practice and Cases in Emergency Medicine* accepts exclusively case reports, images, and clinicopathologic cases; so the chance of successful acceptance is profoundly different between journals. Additionally, you should briefly review several recent issues to determine both the methodological rigor and topics typically accepted.

Read the scope and mission statements of the journals to see if your paper fits. Aligning with the journal’s interests will foster a stronger cover letter when submitting and increase the likelihood of acceptance. There are many subspecialty journals related to EM that focus on specific arenas (e.g., administration, behavioral emergencies, cardiac care, critical care, medical education, prehospital medicine, injury prevention, neuroscience, pediatrics, public health, prehospital care, toxicology, trauma, and ultrasound). If your paper deals with one of these areas, consider expanding your potential submission list to include the relevant subspecialty journals.

Often, several journals will be a good fit for the article, and you must choose. One of the first determinants should be whether the journal is indexed in one of the United States National Library of Medicine’s (NLM) databases. This information is located in the accompanying online table. Alternatively, you can type the name of the journal in the NLM catalog of journals referenced in the National Center for Biotechnology Information Database (PMC; https://www.ncbi.nlm.nih.gov/nlmcatalog/journals/) to determine if the journal is indexed in PubMed or MEDLINE. Currently, 89 titles appear for the search term “emergency medicine.” However, many of these are listed as “not currently indexed in MEDLINE.” This may indicate that the journal is either new, well established but not yet accepted for inclusion, or “predatory.” Importantly, if a journal is not indexed in any of these databases listed in the supplemental table, it has not yet passed the rigorous vetting process of an established journal. You should, therefore, be cautious about submitting your paper there.

If the prospect journal is “open access,” check to see if the journal content is included (i.e., archived) in PubMed Central (https://www.ncbi.nlm.nih.gov/pmc), the NLM’s repository of full research papers. PMC currently contains 2,920 journal titles. Type the journal name into the “*Search for Journals*” box located under PMC Journals (https://www.ncbi.nlm.nih.gov/pmc/journals/) to see if the journal comes up, or you can browse the journal titles through the alphabetical list tabs. If the journal is found, this indicates it has gone through a moderate, multilevel vetting process that typically requires two years of publication and 25–50 submitted papers.

If a journal is in neither of these indices (PubMed or PMC), this may be a reflection of a lesser quality or newer publication. Quality subscription journals are commonly included in PubMed within 5–10 years of inception, and PMC within 2–3 years. Many newer journals are still developing the quality to achieve acceptance to these indices, so they may become PubMed indexed in the coming years. If so, it is customary for previous papers published in the journal before inclusion, to eventually be entered into these indices.

Additional factors to consider when submitting include the journal’s impact factor, InCites Journal Citation Reports^®^, CiteScore^TM^, and Eigenfactor^®^ (discussed further in a subsequent paper in the series). Selecting journals with a higher rating suggests that the article will have more visibility and, therefore, be more likely to be cited. This is important because journal ranking and the number of citations is highly valued by promotion and tenure committees. In general, there is an inverse correlation between a journal’s impact factor and its acceptance rate.

Once you’ve made a list of potential journals, rank them using the above criteria and submit to the top-listed and most relevant journal first. Often, this will be the most rigorous and may result in an early rejection. However, if selected appropriately, the article will be sent out for reviews, which can provide valuable feedback and insights even if the article is rejected.^32^ In some cases, the article may get rejected several times, requiring submission to multiple different journals. When this happens, it is essential to use the feedback from each review to strengthen the article for the next submission.

## DISCRIMINATING BETWEEN LEGITIMATE AND PREDATORY OPEN ACCESS JOURNALS

“Open access” refers to a type of scholarly publication where the author retains the copyright to the work, and access to the entirety of the work is free of charge to readers and other researchers. Typically, the author pays the publisher for their services, with fees ranging from $400 to $4,000 per paper. Legitimate open-access publishers perform substantial scientific peer review with associated detailed revisions prior to publication, and have achieved wide indexing, so that your work can be easily read and cited.

Subscription-based publishers (e.g., Wiley, Blackwell, Taylor & Francis, Elsevier, Springer, Sage, Wolters-Kluwer) require the author to sign over the copyright of their work to the journal in exchange for publication. Authors must subsequently ask the publisher for permission to reproduce any parts of their paper (e.g., table or figure) and publishers often charge a fee for this. Because the publishing services are expensive, rather than charging a fee, the author pays for the services using their scholarly product as payment, and the publisher generates revenue through library subscriptions, copyright sales, and advertising.

Conversely, so-called “predatory” open-access publishing is an exploitative model that involves charging publication fees to authors without providing any significant editorial or publishing services. Predatory journals often identify authors from prior publications or large databases of physicians and routinely solicit submissions by email. They promise rapid review and publication in time frames that preclude substantive peer review.

While papers published by a predatory publisher are, in theory, accessible by other scholars, they do not return in the important indexing service searches that qualified scholars use to find and cite your work. These publishers (more than 900 worldwide in 2017) profit from inexperienced or desperate authors by charging exorbitant publication fees without providing the customary publishing services. Some of these publishers ask authors to provide substantial fees to withdraw their submission during the review period, once the authors realize they have been deceived. Tables 3 and 4 outline the criteria for spotting predatory, open access journals. For those interested in learning more, Hansoti and colleagues provide an excellent review on this topic.^7^

## SURVIVING THE PEER REVIEW PROCESS

Peer review is the backbone of scientific publishing. At its best, it will provide multiple, detailed, independent, and unbiased assessments of your work by clinicians and research peers. This is intended to improve your work prior to dissemination for future use by readers and scientists. Knowing that once published, your work will need to stand alone for years to come should change your perspective to one of welcoming the most thorough critiques in the hope of identifying all flaws prior to public dissemination.

Responding to reviewer critiques can be one of the most important aspects of the manuscript preparation, as it can determine whether your revised manuscript is accepted or rejected. Junior faculty submitting manuscripts for the first time can often feel quite overwhelmed by how to proceed with the critiques due to the number of requests, possible strong tones from reviewers, and the challenge of consolidating disagreements between critiques from different reviewers.

Here are some general principles to consider as you approach revisions and respond to critiques. First, disagreeing or not being able to comply with reviewers, although not preferable, is quite acceptable. However, this decision needs to be factually-based, polite without added emotion, professional, and appropriately referenced.^33^ It might be necessary to mention that a particular revision request is beyond the scope of this research project and justify why this is true. It is particularly important to respond to all of the editor’s comments, which are typically listed first in most journal response letters, though they may be hidden within the general resubmission requirements in some responses. It is in your best interest to acknowledge and appreciate the reviewer and the editor for the time and effort they have provided to improve your work.^34^

It can be valuable to wait 1–2 days prior to responding to let any strong emotions pass and allow you to focus on the scientific components of the paper. When responding to comments, you should make sure to respond to every critique, even if you disagree. This can be facilitated by separating reviewer paragraphs into separate points, listing them in order, and then sequentially responding to each comment.^34^ This response is commonly referred to as a “point-by-point” response. When there is concern regarding how best to approach a comment, or if two reviewer comments contradict each other, it is best to discuss this directly with the editor prior to resubmission. Most journals will provide either the editor’s email or submission query information to assist you.

When submitting the point-by-point response, it can be helpful to highlight your response in a different font style, indentation, or color. Make note of the response, corresponding line numbers, and the verbatim changes you have made in the paper for each comment. Make it as easy as possible for the reviewer and editor to know how you have addressed the request and the exact changes you have made in each specific instance.^33^ Some journals may require you to copy-and-paste the response into their manuscript management system, which would negate the formatting changes noted above. If this is the case, you should also upload a copy of your formatted response appended to the revised cover letter.

If there are concerns regarding grammar or spelling in the manuscript (especially among authors who are less fluent in the submission language), you should consider having an experienced writer or professional copy editor review it to correct all language mistakes. Finally, make sure to review the journal’s revision requirements, as some require submission of manuscripts with tracked changes in the document. Pay attention to the time frame required for revisions, which can be as short as a month. If you cannot meet the deadline, make sure to contact the editor early to ask for an extension. In general, it is best to resubmit as soon as feasible, ideally within one month.

## CONCLUSION

This paper reviews four common challenges faced by all faculty and researchers when writing and publishing their academic work, and provides advice for effectively navigating this arena. We hope that this series will assist junior faculty, fellows, and residents as they pursue successful research and academic careers.

## Supplementary Information



## Figures and Tables

**Table 1:** Top 10 reasons why manuscripts were rejected in Academic Medicine.^19^

Inappropriate or incomplete statistics Overinterpretation of the results Inappropriate or suboptimal instrumentation Sample too small or biased Text difficult to follow Insufficient problem statement Inaccurate or inconsistent data reported Incomplete, inaccurate, or outdated review of the literature Insufficient data presented Defective tables or figures

**Table 2:** ICMJE Authorship criteria.

The ICMJE recommends that authorship be based on the following four criteria: Substantial contributions to the conception or design of the work; or the acquisition, analysis, or interpretation of data for the work; AND Drafting the work or revising it critically for important intellectual content; AND Final approval of the version to be published; AND Agreement to be accountable for all aspects of the work in ensuring that questions related to the accuracy or integrity of any part of the work are appropriately investigated and resolved.

*ICMJE*, International Committee of Medical Journal Editors.

**Table 3:** Criteria for determining the legitimacy of an open access journal.

To determine if an open-access journal is legitimate, look for the following criteria: Search the Directory of Open Access Journals (https://doaj.org/) to see if the journal is listed. Ensure that the journal follows the Committee on Publication Ethics (COPE) standards (https://publicationethics.org/). Ensure that the journal is a member of the International Association of Scientific, Technical, and Medical Publishers (http://www.stm-assoc.org/). Ask colleagues if they are familiar with the journal and determine who else has published in it. Ask your university librarian for guidance. The article processing fee should be transparent and easily found on the journal’s website. The journal’s website should have common policies posted (e.g., conflict of interest, human and animal subjects, plagiarism, informed consent, copyright and authorship, creative commons license type). The Editor-in-Chief and editorial board should be clearly identified with appropriate academic credentials and affiliations. Beware that some predatory journals list editorial board members on their website without the members’ knowledge. Determine whether there is a discount or waiver policy for junior authors or those from low- to middle-income countries or institutional subscriptions.

**Table 4:** Features of a predatory journal.

Grammatical errors in the solicitation or website Unclear or difficult to locate article processing fees Excessively broad and unrelated journal title Impact factor of greater than 2 in an unknown journal Sends out frequent “spam” emails asking for submissions Promise of rapid turnaround to publication (ie, 2 weeks or less) Email addresses from public domain (e.g., Gmail, Yahoo) Western street address with poor grammar or syntax Overly flattering or flowery salutations including: “esteemed author,” “with much greetings and respect,” “kindly participate by submitting…” No mention of indexing beyond Google Scholar No sponsorship by a known medical society Poor quality prior submissions
